# Hydrogen Sulfide (H_2_S)-Releasing Compounds: Therapeutic Potential in Cardiovascular Diseases

**DOI:** 10.3389/fphar.2018.01066

**Published:** 2018-09-21

**Authors:** Lei Zhang, Yanan Wang, Yi Li, Lingli Li, Suowen Xu, Xiaojun Feng, Sheng Liu

**Affiliations:** ^1^The First Affiliated Hospital of USTC, Division of Life Sciences and Medicine, University of Science and Technology of China, Hefei, China; ^2^Aab Cardiovascular Research Institute, University of Rochester, Rochester, NY, United States

**Keywords:** H_2_S, cardiovascular diseases, cardiac hypertrophy/heart failure, ischemia reperfusion injury, atherosclerosis, donor

## Abstract

Cardiovascular disease is the main cause of death worldwide, but its pathogenesis is not yet clear. Hydrogen sulfide (H_2_S) is considered to be the third most important endogenous gasotransmitter in the organism after carbon monoxide and nitric oxide. It can be synthesized in mammalian tissues and can freely cross the cell membrane and exert many biological effects in various systems including cardiovascular system. More and more recent studies have supported the protective effects of endogenous H_2_S and exogenous H_2_S-releasing compounds (such as NaHS, Na_2_S, and GYY4137) in cardiovascular diseases, such as cardiac hypertrophy, heart failure, ischemia/reperfusion injury, and atherosclerosis. Here, we provided an up-to-date overview of the mechanistic actions of H_2_S as well as the therapeutic potential of various classes of H_2_S donors in treating cardiovascular diseases.

## Introduction

Hydrogen sulfide (H_2_S) is a colorless, smelly water soluble gas (Wang, 2010). It was first described in the 17th century (Wang, 2012). Later in 1989, H_2_S was first discovered in rat brain (Altaany et al., 2013). H_2_S is considered to be the third most important endogenous gas molecule in the organism after carbon monoxide (CO) and nitric oxide (NO) (Wang, 2002; Hartle and Pluth, 2016; Szabo, 2016). It can be synthesized in mammalian tissues and can freely cross the cell membrane and exert many biological effects in various systems (Wang, 2012). Studies have found that H_2_S has played a role in neurophysiology, cardiovascular disease, endocrine regulation, and other physiological and pathological processes (Szabo, 2012; Li et al., 2015a).

Hydrogen sulfide is mainly produced by cystathionine gamma-lyase (CSE) and cystathionine beta-synthase (CBS) from L-cysteine and homocysteine (Chiku et al., 2009). It can also be produced in the presence of alpha-ketoglutarate by PRP-independent 3-mercapto-pyruvate sulfate transferase (3-MST) or cysteine aminotransferase (CAT) (Kabil and Banerjee, 2010; Li L. et al., 2011). Free H_2_S can be oxidized by sulfhydryl reductase (SQR) in mitochondria, and it can be methylated by sulfhydryl-*S*-methyltransferase in the cytoplasm (Bouillaud and Blachier, 2011; Levitt et al., 2011). In addition, free H_2_S is excreted through biological fluids after it is combined with methemoglobin and molecules with metal or disulfide bonds (Yang et al., 2004). In the human cardiovascular system, CSE is the major H_2_S production enzyme (Hosoki et al., 1997; Zhao et al., 2001), but the main H_2_S-producing enzyme in rat coronary arteries is 3-MST (Gadalla and Snyder, 2010; Li L. et al., 2011).

H_2_S levels may be enhanced *in vivo* with conventional inorganic sulfide salts, organic H_2_S donors, or phosphodiesterase inhibitors (Bełtowski, 2015). Common H_2_S donors include: sodium hydrosulfide, P-(4-methoxyphenyl)-p-4-morpholinodithiophosphoric acid (GYY4137) (Bankhele et al., 2018); 4-carboxyphenyl-isothiocyanate acid esters (4CPI) (Testai et al., 2016); SG-1002 (Kondo et al., 2013); cysteine analogs; *S*-propylcysteine; *S*-allylcysteine; *N*-acetylcysteine, and other drug chimeras such as L-DOPA, NOSH-sulindac (AVT-18A), NOSH-aspirin, ACS67 (mixed compound of latanoprost and H_2_S releasing moiety) (Kashfi and Olson, 2013; Salvi et al., 2016a). Among them, *S*-propargyl-cysteine (SPRC), which can slowly release H_2_S, also called ZYZ-802, is an analog of *S*-allylcysteine (SAC), and SAC is the most abundant component in aged garlic extract (Wen and Zhu, 2015). *N*-acetylcysteine (NAC) is commonly used as an antioxidant and cell protectant (Cerda and Pluth, 2018). L-cysteine is a substrate for the endogenous production of H_2_S (Salvi et al., 2016b), mitochondria-targeted anethole dithiolethione (AP39), and (AP123) (Gerő et al., 2016). Please see **Table 1** for more information on common H_2_S donors as well as CBS and CSE inhibitors (Lertratanangkoon et al., 1999; Lima et al., 2006; Szabó, 2007; Li et al., 2008; Kulkarni et al., 2009; Sun et al., 2009; Tyagi et al., 2009; Liu et al., 2010, 2011; Wang et al., 2010, 2016; Kodela et al., 2012; Predmore et al., 2012; Guo et al., 2013; Kondo et al., 2013; Lisjak et al., 2013; Luo et al., 2013; Martelli et al., 2013, 2014; Barr et al., 2015; Fonseca et al., 2015; Iciek et al., 2015, 2016; Liang D. et al., 2015; Polhemus et al., 2015; Tsai et al., 2015; Vannini et al., 2015; Chao et al., 2016; Qian et al., 2016; Salvi et al., 2016a; Tain et al., 2016; Testai et al., 2016; Wu et al., 2016; Ali et al., 2018; Cao et al., 2018; Du et al., 2018; Ezeriņa et al., 2018; Liang et al., 2018; Lin S. et al., 2018; Ning et al., 2018; Powell et al., 2018; Qiu et al., 2018; Shimizu et al., 2018; Sone et al., 2018; Zhao et al., 2018; Zhou et al., 2018).

**Table 1:** Structure and characteristics of common H_2_S donors and inhibitor of CSE or CBS.

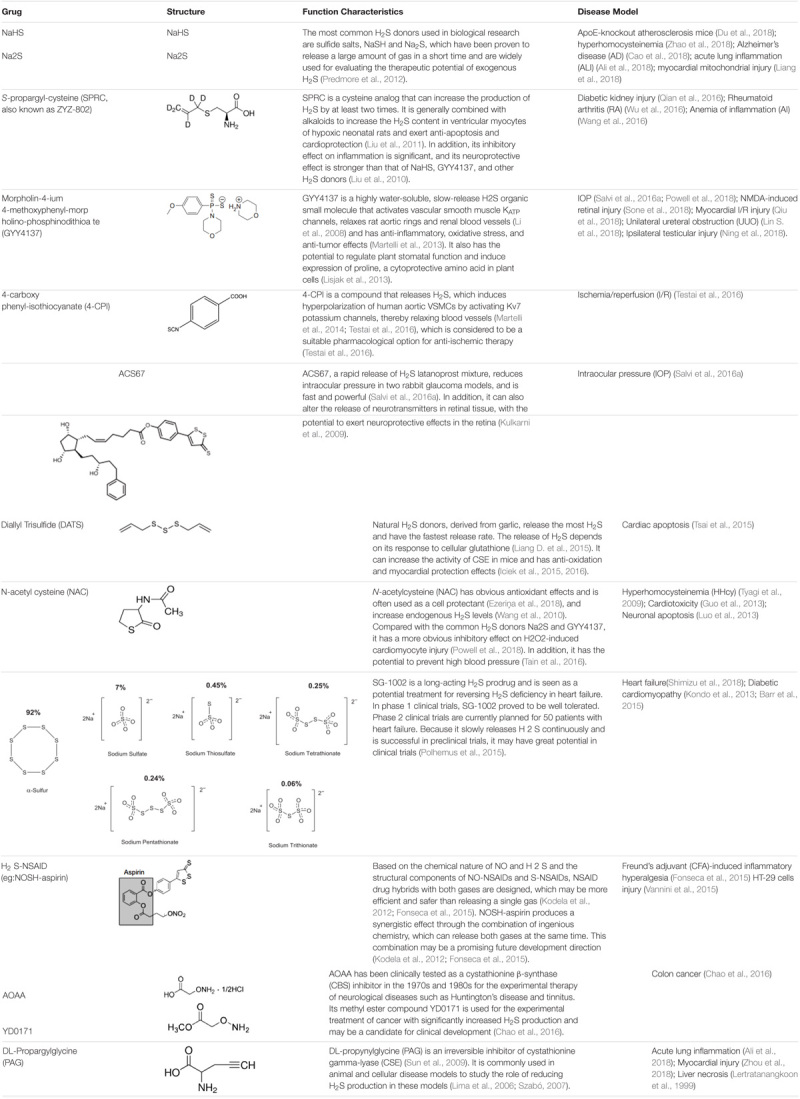

To date, there has been a lot of research on the therapeutic effects of H_2_S in atherosclerosis, cardiac remodeling, and myocardial ischemia-reperfusion injury (Yang et al., 2008; Wang et al., 2009; Calvert et al., 2010; Xu et al., 2014). Its related mechanisms involve anti-oxidation, inhibition of cell apoptosis, pro-angiogenesis, anti-inflammatory, ion channel regulation, and so on (Wang, 2012; Xu et al., 2014).

## H_2_S and Cardiovascular Diseases

### Atherosclerosis

Atherosclerosis is based on lipid metabolism disorder and is the main cause of various cardiovascular and cerebrovascular diseases, including coronary heart disease, cerebral infarction, and peripheral blood vessels. The development of atherosclerosis involves a variety of mechanisms, including endothelial cell damage and dysfunction, inflammatory cell recruitment, foam cell formation, smooth muscle cell proliferation and migration, calcification, fibrous cap rupture, and thrombosis (Bentzon et al., 2014; Nowak et al., 2017). Atherosclerotic lesions start from the intima, usually with accumulation of lipids and complex carbohydrates, hemorrhage and thrombosis, and then fibrous tissue hyperplasia and calcinosis, and gradually gradual metamorphosis and calcification of the arterial layer, leading to thickening of the arterial wall hardened, narrowed blood vessel lumen (Bentzon et al., 2014; Escárcega et al., 2018; Raggi et al., 2018).

Diabetes mellitus (DM) is a metabolic disease characterized by hyperglycemia due to defects in insulin secretion and/or impaired biological effects. Hyperglycemia, which persists in diabetes, can cause chronic damage, endothelial dysfunction, and atherosclerosis (Bai et al., 2018; Wu T. et al., 2018).

#### Deregulation of H_2_S and H_2_S Producing Enzymes in Human and Mouse Atherosclerosis

Plasma H_2_S levels were obviously lower in patients with acute coronary syndrome (ACS), compared with patients with non-coronary artery disease (CAD), or stable angina pectoris (SAP). The levels of plasma monocyte chemokine receptor CCL2 and CX3CL1 were significantly increased (Gao et al., 2015b). In addition, studies in 113 patients with chronic hemodialysis showed an increase in cardiovascular risk factors (such as atherosclerosis) and mortality, which may be related to low levels of plasma H_2_S, activation of PKCβII, and upregulation of VCAM-1/ICAM-1 (Feng et al., 2015). The plasma H_2_S and aortic H_2_S levels were decreased in ApoE^(-/-)^ mice. CSE expression was reduced in oxidized LDL (Ox-LDL)-stimulated human aortic endothelial cells (HAEC) and in the aorta of high fat diet-induced ApoE^(-/-)^ mice (Leucker et al., 2017). The infiltration of red blood cells into atherosclerotic plaques is associated with atherosclerosis. Inside the lesion, hemoglobin (Hb) is oxidized to ferrous and sulfhydryl Hb to exhibit pro-oxidative and pro-inflammatory activity. The expression of CSE is mainly up-regulated in macrophages, foam cells, and myofibroblasts from human atherosclerotic lesions of patients with carotid specimens. A similar pattern was observed in aortic lesions of ApoE^(-/-)^ mice on a high-fat diet. H_2_S can obviously reduce the oxidation of Hb and inhibit the progression of atherosclerosis (Potor et al., 2018). In mouse and human atherosclerosis, CSE expression is upregulated, but circulating and plaque levels of H_2_S are reduced, a phenomenon that can be attributed to inhibition of CSE enzyme activity (Bibli et al., 2018).

In adipose tissue macrophages (ATM) isolated from diet-induced obese mice, the intracellular concentration of H_2_S was lower than that of H_2_S in ATM from lean mice. The intracellular H_2_S concentration in the mouse macrophage cell line RAW264.7 was decreased during the inflammatory reaction induced by lipopolysaccharide (LPS). Production of pro-inflammatory cytokines in RAW264.7 cells and ATM from obese mice can be inhibited by exogenous H_2_S (Velmurugan et al., 2015). Homocysteine (Hcy) is a precursor of H_2_S, which forms H_2_S by a transsulfide pathway catalyzed by CBS and CSE (Li et al., 2015b). Hcy inhibits CSE expression by increasing DNA methylation in the CSE promoter region, whereas DNA methyltransferase (DNMT) knockout reverses the reduction of CSE transcription in Hcy-induced macrophages (Li et al., 2015b; Du et al., 2016). MicroRNAs (miRNAs) are small, siRNA-like molecules encoded by the genome of higher eukaryotes and are approximately 22 nucleotides in length. The miRNA induces silencing complex (RISC) to degrade mRNA or hinder translation by base pairing with the target gene mRNA (Bartel, 2009). CSE/H_2_S obviously increased ATP-binding cassette transporter A1 (ABCA1) expression and regulated cholesterol efflux in human THP-1 macrophage-derived foam cells, and MiR-216a significantly reduces CSE expression by directly targeting its 3′ untranslated region, thereby increasing cholesterol levels in THP-1 macrophage-derived foam cells (Gong et al., 2016). Similarly, MiR-186 can also directly inhibit CSE expression by targeting its 3′ untranslated region to promote THP-1 macrophage pro-inflammatory cytokine secretion and lipid accumulation (Yao et al., 2016).

#### H_2_S Donors Prevents Atherosclerosis *in vivo*

Endogenous H_2_S production is increased in aortic tissues of ApoE knockout and CSE gene overexpression mice (Tg/KO), atherosclerotic plaque size is reduced, and plasma lipid profile is reduced. Thus, activation of the CSE gene attenuates atherosclerotic symptoms in ApoE^(-/-)^ mice (Cheung et al., 2014). GYY4137 reduced aortic atherosclerotic plaque in ApoE^(-/-)^ mice and improved aortic endothelium-dependent relaxation. The specific mechanism is that GYY4137 reduces the expression of aortic ICAM-1, TNF-α, IL6, and LOX-1, and increases eNOS phosphorylation and PI3K expression (Liu et al., 2013). NaHS reduces atherosclerotic plaque in atherosclerotic rats and reduces ET-1 production in rat aortic endothelium (Liu et al., 2013). H_2_S can exert its cytoprotective effect through cysteine *S*-thiol to scavenge free radicals and inhibit oxidative stress, thereby inhibiting atherosclerosis (Cheung and Lau, 2018). In a mouse model of atherosclerosis induced by partial ligation of the left common carotid artery (LCA), NaHS administration significantly reduced the severity of atherosclerosis. This may be due to H_2_S up-regulating the expression of angiotensin converting enzyme 2 (ACE2) in the carotid artery, thereby converting angiotensin II to angiotensin 1-7 (Lin et al., 2017). H_2_S can also inhibit atherosclerosis in ApoE^(-/-)^ mice as well as the proliferation and migration of vascular smooth muscle cells (VSMCs) by upregulating plasma NO (Lin et al., 2016). High-fat diet induced a significant decrease in plasma H_2_S levels and atrial natriuretic peptide (ANP) levels in rats with atherosclerosis, and elevated adrenomedullin (ADM) levels. Treatment with NaHS for 8 weeks reversed these changes in atherosclerotic rats (Li et al., 2015c). NaHS can also up-regulate the expression of ABCA1 by promoting the nuclear translocation of PPARα, which significantly reduces serum triglyceride (TG), cholesterol (TC), low-density lipids protein (LDL) levels, and atherosclerotic plaque size in high-fat diet-fed ApoE^(-/-)^ mice (Li et al., 2016). SHR rats exhibit vascular remodeling and collagen accumulation. H_2_S regulates vascular collagen and inhibits VSMCs proliferation and collagen production (Zhao et al., 2008).

*S*-sulfuration is a signaling pathway for H_2_S, which is thought to be an antiatherogenic molecule that can prevent atherosclerosis. H_2_S induces *S*-sulfuration of glutathione peroxidase 1 and further reduces lipid peroxidation and increases antioxidant defense in the aorta by promoting glutathione synthesis (Cheung and Lau, 2018). NaHS or GYY4137 reduces SIRT1 degradation by direct s-sulfhydration, thereby reducing atherosclerotic plaque area, macrophage infiltration, aortic inflammation, and plasma lipid levels in ApoE^(-/-)^ mice (Du et al., 2018). H_2_S can reduce aortic atherosclerotic plaque formation in streptozotocin (STZ)-treated LDLr^(-/-)^ mice, but not LDLr and Nrf2 double knockout mice. This inhibitory effect of H_2_S may be related to Nrf2 activation through Keap1 s-sulfhydration (Xie et al., 2016). In oscillatory shear stress (OSS)-induced atherosclerosis, CSE expression is down-regulated, and NaHS activates eNOS and decreases the expression of intercellular ICAM-1, thereby inhibiting OSS-promoting atherosclerosis (Go et al., 2012). Estrogen can increase the production of H_2_S in the liver and vascular tissue by increasing the activity of CSE, thereby inhibiting atherosclerosis in female mice (Li et al., 2017a). Similarly, the estrogen 17β-estradiol (E2) activates CSE/H_2_S through PKG in endothelial cells, thereby dilating blood vessels and attenuating atherosclerosis in mice (Zhou et al., 2013).

VSMC plays a significant role in diseases such as atherosclerosis and restenosis after invasive intervention. S-Diclofenac is a novel molecule containing H_2_S (H_2_S is linked to diclofenac via an ester bond), inhibits smooth muscle cell proliferation, and may play a role in restenosis in vascular injury sites (Baskar et al., 2008). Similarly, atherosclerotic lesions were induced in rabbits, and we treated rabbits in a similar manner to balloon angioplasty (BA). NaHS treatment significantly reduced VSMCs proliferation in the neointimal, while DL-propargyl glycine (an inhibitor of H_2_S synthase) significantly induced VSMCs proliferation. Thus, H_2_S attenuates neointimal hyperplasia and inhibits restenosis after BA (Kuiper et al., 2001). In ApoE^(-/-)^ mice, H_2_S inhibits proliferation and migration of VSMCs and inhibits the development of atherosclerosis by increasing plasma NO levels and increasing levels of *S*-nitrosylated proteins in VSMCs (Lin et al., 2016). Farnesyl pyrophosphate synthase (FPPS) plays an important role in the mevalonate pathway, and the FPPS inhibitor alendronate can alleviate diabetes-induced atherosclerosis and inhibit high glucose-induced proliferation of VSMCs *in vitro*. Specific mechanisms may be through reducing H_2_S metabolism and inhibiting small GTPase (Rac1, RhoA and Ras) activities (Chen et al., 2017).

#### H_2_S Donors Prevents Atherosclerosis *in vitro*

##### Improving endothelial dysfunction

Endothelial dysfunction is a vital event event in the early stages of atherosclerosis (Peng et al., 2017). Hyperglycemia is a key factor in the development of diabetic complications, such as atherosclerosis (Lin J. et al., 2018). Receptor interacting protein 3 (RIP3) mediates necrotic apoptosis and is involved in the development of atherosclerosis. NaHS significantly attenuated high-glucose (HG)-induced apoptosis of HUVECs by inhibiting the expression of RIP3 (Lin J. et al., 2018). NaHS also reduces atherosclerotic plaque in rats by protecting vascular endothelial cells and reducing the production of aortic endothelium ET-1 (Li et al., 2015d). In HUVEC, H_2_S inhibits H_2_O_2_-mediated mitochondrial dysfunction by maintaining levels of intracellular antioxidant enzymes (Go and Jones, 2005). In HUVEC, NaHS promotes expression of eNOS protein and NO production by increasing the expression of miR-455-3 (Li et al., 2017b). Similarly, in HUVEC, H_2_S inhibits TNF-α stimulated ICAM-1 expression by inhibiting NF-κB pathway (Wang et al., 2009). Two new mitochondrial targeting H_2_S donors AP39 and AP123 (30–300 nM) inhibit HG-induced damage by reducing hyperpolarization of endothelial cell mitochondrial membranes and inhibiting mitochondrial oxidant production. These mitochondria-targeted donors have an >1000-fold increase in potency in inhibiting HG-induced endothelial damage. This suggests that these compounds are useful for combating diabetic vascular complications (Gerő et al., 2016). Under the condition of glucose oxidase-induced oxidative stress, endothelial cells have enhanced oxidative stress, inhibited cell bioenergetic function, and decreased cell viability. AP39 pretreatment significantly attenuated the above reaction (Szczesny et al., 2014).

Tet methylcytosine dioxygenase 2 (TET2) is a DNA demethylase. In human umbilical vein endothelial cells (HUVECs), oxLDL treatment down-regulates TET2 and CSE/H_2_S, whereas TET2 overexpression up-regulates CSE/H_2_S by DNA demethylation of the CSE gene promoter, thereby inhibiting oxLDL stimulated NF-κB activation and ICAM-1 expression (Peng et al., 2017). Zofenoprilat is an active metabolite of zofenopril, which inhibits interleukin-1β (IL-1β)-induced inflammatory responses in HUVECs via the CSE/H_2_S pathway (e.g., NF-κB/COX-2 activation) (Monti et al., 2016). Its inflammation inhibition is also verified in vascular smooth muscle cells and fibroblasts (Monti et al., 2016). In addition, the HDAC6 inhibitor tubacin and HDAC6-specific siRNA inhibited OxLDL-induced decrease in endothelial cell CSE expression and improved endothelial function (Leucker et al., 2017).

##### Improving VSMCs dysfunction

CSE knockout (CSE-KO) mice’s mesenteric artery VSMCs, compared with CSE-WT cells, CSE-KO cells showed redox imbalance and abnormal mitochondrial activity, and were also more sensitive to hypoxia-induced cell death. It indicates that the endogenous CSE/H_2_S pathway significantly regulate the normal function of VSMCs (Bryan et al., 2011). Insulin-like growth factor-1 (IGF-1) exerts a variety of physiological and pathophysiological effects on the vascular system, including stimulation of VSMCs proliferation and migration. For VSMCs isolated from mesenteric arteries of wild type and CSE knockout mice, IGF-1 increases the proliferation of VSMCs, and the effect is more pronounced in CSE knockout-VSMCs. In addition, H_2_S significantly down-regulates IGF-1R expression, stimulates IGF-1R S-sulfation, and impairs IGF-1 binding to IGF-1R, thereby inhibiting IGF-1-induced VSMCs proliferation (Shuang et al., 2018). In VSMCs, H_2_S significantly reversed the decrease in iNOS expression and NO production induced by oxidized low-density lipoprotein and increased the protein *S*-nitrosylation level of VSMCs (Lin et al., 2016).

T-type channels (Cav3.1, 3.2, and 3.3) obviously affect the proliferation of VSMCs and H_2_S selectively inhibits T-channel Cav3.2. H_2_S induces concentration-dependent proliferation inhibition in the human coronary artery smooth muscle cells and smooth muscle cell line A7r5, however, this mechanism of suppressing cell proliferation is independent of selective inhibition of T-type channels by H_2_S (Elies et al., 2015). H_2_S reduces myogenic tension and causes relaxation of the phenylephrine (PE) mesenteric artery. H_2_S relaxes blood vessels by activating the large-conductance Ca^(2+)^-activated potassium channel (BKCa) and Cyp2C, a novel vasodilation pathway of emerging signaling molecules (Jackson-Weaver et al., 2013). H_2_S can also dilate blood vessels by increasing the K_ATP_ channel opening in VSMCs (Tang et al., 2005). In addition, maintaining cardiovascular homeostasis requires normal arterial baroreflex, and its sensitivity decreases during vascular calcification (VC). H_2_S can promote baroreflex sensitivity in hypertensive rats. H_2_S can directly promote the damage of baroreceptors in VSMCs calcification VC rats and improve VC (Li et al., 2017c). Vitamin D3 plus nicotine (VDN) induces VC and phenotypic conversion of VSMC in rats. H_2_S may alleviate VC and VSMC phenotypic changes by reducing endoplasmic reticulum stress (ERS) (Yang et al., 2016). In addition, NaHS enhances the expression of NADPH dehydrogenase 1 (NQO1) by enhancing nuclear factor (erythrocyte-derived 2)-like 2 (NRF2) activity, which *in vitro* attenuates calcineurin-induced calcification of VSMCs by cyclic troponin particles (CPP) (Aghagolzadeh et al., 2017).

##### Improving macrophage dysfunction

Cystathionine gamma-lyase expression and H_2_S production were reduced in oxidized ox-LDL-stimulated macrophages. Overexpression of CSE decreased ox-LDL-induced TNFα production by inhibiting the JNK/NF-κB signaling pathway (Wang et al., 2013). The NADPH oxidase member NADPH oxidase 4 (Nox4) is closely related to the production of reactive oxygen species (ROS). CSE knockdown expands inflammation by up-regulating Nox4-ROS signaling in sepsis mice and macrophages, and CSE overexpression reduces macrophage-enhanced inflammatory mediator production (Wang et al., 2018). GYY4137 inhibited the increase in TNF-α and IL-1β in HHcy mouse plasma and Hcy-stimulated RAW264.7 cells, whereas the CSE inhibitor PAG aggregated it (Li et al., 2015b). RAW264.7 or mouse peritoneal macrophages were stimulated with NaHS or saline and then induced by interferon-gamma (IFN-γ) or LPS. NaHS obviously attenuated the expression of aortic CX3CR1 and CX3CL1, as well as aortic plaque by modulating proliferator-activated receptor-gamma (PPAR-γ) and NF-κB activities (Zhang H. et al., 2012). The novel slow release H_2_S release compound FW1256 reduces the levels of TNFα, IL6, PGE, and NO in LPS-stimulated RAW264.7 macrophages (Huang et al., 2016).

Hypoxia-inducible factor 1-alpha (HIF-1α) plays an important regulatory role in inflammation. In THP-1 macrophages, H_2_S activates the HIF-1α/nuclear factor E2-related factor 2 (Nrf2) signaling pathway through the p38-MAPK pathway to reduce inflammation (Lohninger et al., 2015). H_2_S inhibits ox-LDL-stimulated macrophage inflammation response by inhibiting NF-κB recruitment to the monocyte chemoattractant protein 1 (MCP-1) promoter. Its molecular mechanism may be the s-sulfhydration of p65 (Du et al., 2014). In macrophages, *S*-sulfation of c-Jun by H_2_S enhances its transcription of p62 and SIRT3, which leads to a reduction in mtROS production and NLRP3 inflammasome activation (Lin et al., 2018). In addition, the absence of glutathione (GSH) and the upregulation of IL-1β are associated with the progression of vascular inflammation and atherosclerosis. H_2_S up-regulates GSH and suppresses IL-1β in U937 monocytes (Jain et al., 2014). H_2_S also inhibits histone acetylation in macrophages induced by LPS and inhibits transcription of various pro-inflammatory cytokines (Rios et al., 2015). The Jumonji domain protein 3 (JMJD3) is a histone demethylase with a target of histone 3 Lys27. JMJD3 knockdown attenuates LPS-induced inflammatory responses. Over-expression of CSE can reduce the inflammatory mediators produced by macrophages and thereby attenuate LPS-induced inflammatory responses by regulating JMJD3 expression in sepsis mice (Liu et al., 2018). Foam cell formation is a hallmark of atherosclerosis (Xu et al., 2011). H_2_S supplementation also inhibits foam cell formation in macrophages stimulated with pro-atherogenic factors, such as HG and oxLDL (Zhou et al., 2014; Liu et al., 2013; Xie et al., 2016).

##### Inhibiting platelet activation and thrombus formation

The relationship between H_2_S and platelet aggregation is not yet clear, and its mechanism of action needs further study. GYY4137 reduces thrombus stability by reducing platelet-leukocyte aggregation, thereby promoting endogenous thrombolysis (Grambow et al., 2017). NaHS may inhibit ADP or thrombin-induced platelet aggregation at least in part by inhibiting gap junctional cell-cell communication, and H_2_S released by H_2_S-releasing aspirin derivative (ACS14) may promote other antiplatelet effects *in vitro* compared to aspirin (Gao et al., 2015a). NaHS can inhibit collagen-induced platelet aggregation, and the mechanism is related to change of platelet [Ca]^2+^ (Zhong et al., 2014). Antithrombotic effects of GYY4137 in mice may modulate thrombosis by interfering with adhesion molecule-induced aggregation and platelet activation (Grambow et al., 2014). NaHS inhibits platelet aggregation in rabbits, which is dependent on cAMP (Nishikawa et al., 2013). H_2_S and dithionite inhibit platelet aggregation stimulated by ADF and collagen (Zaichko and Pentiuk, 2009). NaHS also significantly reversed endothelial cell (EC) damage and platelet aggregation induced by hyperhomocysteinemia (Wang et al., 2017).

In addition, NaHS inhibits platelet aggregation stimulated by collagen, ADP, arachidonic acid, epinephrine, thromboxane mimetics, U46619, and thrombin. However, this study considered that this effect of H_2_S does not depend on NO production, cAMP/cGMP production, or opening of potassium channels (Zagli et al., 2007). The above studies support H_2_S inhibition of platelet aggregation; however, there is also the opposite conclusion: NaHS significantly increases platelet aggregation collected in healthy volunteers induced by peptide-6 amide (a thrombin receptor activator) (d’Emmanuele et al., 2013).

### Cardiac Hypertrophy and Heart Failure

Cardiac remodeling, including progressive pathological changes in heart and vessel size, shape, structure, and function, is characterized by progressive cardiac hypertrophy, ventricular dilatation, cardiac fibrosis, apoptosis, vascular dysfunction, and ultimately heart failure (HF) (Pfeffer and Braunwald, 1990; Rizzello et al., 2009). Prevention or reversal of cardiac remodeling is a key strategy for the treatment of HF (Konstam et al., 2011). The mechanisms of cardiac remodeling are complex, including the renin-angiotensin-aldosterone system (RAAS), autophagy, apoptosis, inflammation, matrix metalloproteinases, miRNAs, transcriptional, and post-transcriptional modifications (Swynghedauw, 1999; Maytin and Colucci, 2002; Fan et al., 2017). To illustrate the role of H_2_S in cardiac remodeling, we describe cardiac remodeling according to its inducing factors.

#### Effect of H_2_S on Cardiac Remodeling Induced by Renin–Angiotensin Receptor Agonists

Hyperstimulation of the β-adrenergic receptor (β-AR), which produces a hypertrophic effect in cardiomyocytes, can rapidly reduce endogenous H_2_S levels. Glucose-6-phosphate dehydrogenase (G6PD) is the rate-limiting enzyme that produce NADPH, and H_2_S may inhibit cardiac hypertrophy induced by adrenergic overstimulation by enhancing G6PD activity (Chhabra et al., 2018). Exogenous H_2_S also inhibits cardiac apoptosis and inhibit isoprenaline (ISO)-induced cardiac remodeling by maintaining mitochondrial membrane potential and reducing ROS production in mitochondria (Lu et al., 2013). In addition, exogenous administration of H_2_S inhibits ISO-induced left ventricular hypertrophy (LVH) by up-regulating CSE mRNA/H_2_S, while reducing systolic blood pressure and pulse wave velocity (Ahmad et al., 2018). ZYZ-802, a novel synthetic HS-NO hybrid molecule that decomposes H_2_S and NO, attenuates ISO-induced heart failure by increasing vascular endothelial growth factor (VEGF) levels and cyclic guanosine 5′-monophosphate (cGMP) levels (Wu D. et al., 2018). H_2_S also improve ISO-induced heart failure by inhibiting mast cell infiltration and renin degranulation to inhibit local renin levels (Liu et al., 2014).

In addition, sodium thiosulfate (STS), a clinically applicable H_2_S donor, and NaHS reduce Ang II-induced hypertension, cardiac hypertrophy, tissue fibrosis, and oxidative stress in rats (Snijder et al., 2015). In neonatal rat cardiomyocytes, H_2_S prevent Ang-II-induced cardiac hypertrophy by activating the Nrf2 pathway and reducing oxidative stress (Shao et al., 2017). H_2_S even improves glucose utilization in cardiomyocytes, including increased glucose uptake and glycolysis and citric acid cycle efficiency, inhibiting phenylephrine-induced cardiomyocyte hypertrophy (Liang M. et al., 2015).

#### Effect of H_2_S on Pressure Overload-Induced Cardiac Remodeling

Krüppel-like factor 5 (KLF5) exerts multiple functions in the cardiovascular system (McConnell and Yang, 2010). KLF5 knockout mice may reduce Ang II-induced inflammatory vascular responses and cardiac hypertrophy (Shindo et al., 2002). GYY4137 regulates KLF5 transcriptional activity through specific protein 1 *S*-sulfhydration to inhibit cardiac remodeling in spontaneously hypertensive rats (Meng et al., 2016). GYY4137 also inhibits cardiac fibrosis in hypertensive rats (SHR) by inhibiting TGF-β1/Smad2 signaling pathways, and inhibiting Alpha-smooth muscle actin (α-SMA) expression in cardiac fibroblasts (Meng et al., 2015b). Animal and human studies have shown that in LVH, changes in the expression of connexin 43 (Cx43) and disorganization of gap junctions are the basis for the occurrence and development of arrhythmia (Danik et al., 2004; Teunissen et al., 2004). H_2_S obviously inhibit cardiac hypertrophy and fibrosis caused by coarctation of the abdominal aorta by reducing the activity of cardiac Ang-II and up-regulating the expression of Cx43 (Huang et al., 2012). H_2_S induces angiogenesis and promotes blood vessel growth in the context of hindlimb ischemia and reduces left ventricular remodeling and dysfunction induced by lateral aortic coarctation in mice by promoting the growth of new blood vessels (Polhemus et al., 2013). SG-1002 inhibits myocardial remodeling induced by transverse aortic constriction in mice by up-regulating the expression of endothelial nitric oxide synthase (eNOS) and the production of NO (Kondo et al., 2013).

CSE knockout mice and heart-specific overexpressed mice were prepared using knock-out or transgenic techniques. After transverse aortic coarctation surgery, cardiac hypertrophy was significantly aggravated in CSE-knockout mice, but cardiac hypertrophy was significantly reduced in overexpressing mice. Mechanism studies have shown that CSE upregulates vascular endothelial growth factor-Akt-eNOS-NO-cGMP pathway, maintain mitochondrial function, weaken oxidative stress, and increase myocardial vascular density (Kondo et al., 2013). In addition, H_2_S improves heart function in chronic heart failure rats induced by coarctation of abdominal aorta by dilating blood vessels and affecting extracellular collagen metabolism (Especially type I collagen) (Li X.H. et al., 2011). H_2_S also induces matrix metalloproteinases (MMP)-2 to enhance VEGF synthesis and angiogenesis, while inhibiting TIMP-3 and MMP-9 levels, reducing anti-angiogenesis factors, and reducing intracardiac fibrosis and cardiac remodeling in pressure-overloaded mice (Givvimani et al., 2011). Heme oxygenase-1 (HO-1) is up-regulated by many oxidative stress in the cardiovascular system (Immenschuh and Schröder, 2006). HO-1 also reduce atherosclerotic lesions, ischemic myocardial injury, and modulated blood pressure (Johnson et al., 1997; Ndisang et al., 2002; Otterbein et al., 2003; Yet et al., 2003; Fujimoto et al., 2004; Liu et al., 2006). H_2_S inhibits volume overload-induced chronic heart failure (CHF) through up-regulation of HO-1 expression (Zhang et al., 2013).

#### Effects of H_2_S on Ischemic Injury-Induced Cardiac Remodeling

Hydrogen sulfide has beneficial effects on left ventricular hypertrophy after myocardial infarction (MI) in mice. H_2_S improves ischemic heart failure in mice by inhibiting oxidation, increasing mitochondrial biogenesis, and reducing apoptosis (Wu et al., 2017). In the heart failure (HF) model after MI induced by left coronary artery ligation in the left anterior descending coronary artery, NaHS inhibits heart cell apoptosis and improve mitochondrial dysfunction in HF hearts (Calvert et al., 2010). SPRC is a new type of endogenous H_2_S controlled-release preparation that protects rat HF from left coronary occlusion by maintaining levels of antioxidant molecules such as GSH, CAT, and SOD (Huang et al., 2013). NaNO_2_ significantly improved ischemia-induced left ventricular function in CHF mice by increasing H_2_S bioavailability, Nrf2 activation, and antioxidant defense (Donnarumma et al., 2016b). In addition, Na_2_S treatment inhibits ischemic heart failure in mice by inhibiting nuclear export of histone deacetylase 4 and apoptotic signaling kinase-1 signaling in a thioredoxin 1 dependent manner (Nicholson et al., 2013). Na_2_S also enhances cardiac proteasome activity and function through Nrf2-dependent manner, thereby inhibiting cardiac dysfunction (Shimizu et al., 2016).

#### The Effect of H_2_S on Other Types of Cardiac Remodeling

The level of H_2_S in mice with diabetic cardiomyopathy was reduced. H_2_S is able to improve the energy metabolism of cardiac tissue in db/db mice by up-regulating the expression and activity of SIRT3 (Sun et al., 2018). H_2_S also relieves streptozotocin-induced diabetic diabetic cardiomyopathy (DCM) development by reducing inflammation, oxidative stress, and apoptosis (Zhou et al., 2015a).

In the high-fat diet (HFD)-induced mouse cardiomyopathy model, circulating and cardiac H_2_S levels are also reduced, and SG-1002 treatment restores adiponectin levels and inhibit cardiac endoplasmic reticulum stress (Barr et al., 2015). Elevated homocysteine levels in hyperhomocysteinemia (HHcy) are the inducing factors of pathological cardiac remodeling. HHcy induces cardiac hypertrophy by facilitating MEF2C-HDAC1 complex formation, inactivating MEF2C, and inhibiting miR-133a in cardiomyocytes. H_2_S inhibits myocardial hypertrophy by activating MEF2C and inducing miR-133a in cardiomyocytes (Kesherwani et al., 2015). There may be negative feedback regulation between CBS and CSE enzymes, and *in vivo* studies using CBS^(±)^ mice show that CBS deficiency increases cardiac CSE (Nandi and Mishra, 2017). In addition, H_2_S donor therapy reduces arteriovenous fistula (AVF)-induced cardiac cell fibrosis and apoptosis in mice by reducing oxidative and proteolytic pressures (Mishra et al., 2010). In AVF-induced heart failure model in mice, sodium thiosulfate (STS) partially improves cardiac dysfunction in mice by increasing H_2_S production (Sen et al., 2008). Passive smoking established rat left ventricular remodeling model. H_2_S may produce anti-oxidation through PI3K/Akt-dependent activation of Nrf2 signaling, thereby reducing ventricular remodeling (Zhou et al., 2015b). H_2_S improves endothelin-induced cardiac hypertrophy and fibrosis in rats (Yang et al., 2014). NaHS stimulates ANP secretion and reduces atrial pressure (AP) in the rat atrium through the K-channel and PI3K/Akt signaling pathway (Yu et al., 2018).

### Ischemia Reperfusion Injury

Ischemic heart disease is mainly caused by atherosclerotic lesions of the coronary arteries, with a reduction in blood supply to the heart. The most serious type is myocardial infarction with a high mortality rate. Reperfusion is necessary to improve ischemia, but it also causes irreversible myocardial damage (Heusch, 2015). Therefore, it is necessary to understand the potential mechanisms of myocardial ischemia-reperfusion design, including AMPK, Akt, MAPK, PKA, and NO (Heusch, 2015, 2017) to better treat myocardial ischemia-reperfusion injury.

There are many reports on the role of H_2_S in cardiac I/R injury in rats. For example, NaHS prevents rat I/R heart damage by reducing the expression of proinflammatory cytokines and inducible nitric oxide synthase and up-regulating Akt/endothelial nitric oxide synthase (eNOS) (Issa et al., 2013). NaHS reduces infarct size of rat heart induced by I/R includes upregulation of heat shock protein 72 (Bliksøen et al., 2008), increased phosphorylated Akt and phosphorylated mTOR (Zhou et al., 2014), inhibition of mitochondria permeability transition (MPT) pore openings and increased cardiac mitochondrial membrane potential (Shymans’ka et al., 2012), and activation of PKC to regulate Intracellular Ca^(2+)^ overload (Pan et al., 2008). Homocysteine levels and endogenous H_2_S production are primarily regulated by CBS and CSE enzymes. NaHS also improved myocardial recovery after cardiac I/R injury, however, its protective effect was abolished in CSE^(-/--)^, CBS^(-/-)^, and dietary hyperhomocysteinemia mice (Nakano et al., 2015). Hydrosulfide inhibits acute myocardial infarction (AMI)-induced apoptotic cell death by enhancing the phosphorylation of GSK-3β and the concentration of β-catenin (Ge et al., 2016). GYY4137 can prevent myocardial infarct size in rats by enhancing PI3K/Akt signaling (Karwi et al., 2016), reducing oxidative stress and apoptosis (Meng et al., 2015a). H_2_S prevents myocardial infarct size in rats by enhancing AMPK activity and restoring I/R impaired autophagy (Xie et al., 2015). H_2_S restores mitochondrial dysfunction, thereby reducing myocardial damage in I/R-impaired rat hearts (Ansari and Kurian, 2016). H_2_S prevent myocardial infarct size in rats by increasing the mitochondrial K_ATP_ channel opening time and the K_ATP_ opening frequency (Zhang Z. et al., 2007; Ji et al., 2008). H_2_S treatment inhibits myocardial infarct size in rats by activating the Akt, PKC, and eNOS pathways (Yong et al., 2008). In addition, H_2_S significantly reduced the I/R infarct size in isolated hearts, decreased the activity of creatine kinase and lactate dehydrogenase in heart tissue, and restored mitochondrial dysfunction (Ravindran et al., 2016). H_2_S inhibits myocardial infarct size and myocardial enzyme release in rats by activating Sirt1/PGC1α and JAK2/STAT3 signaling pathways (Luan et al., 2012; Hu et al., 2016), and by inhibiting oxidation and inhibiting the release of inflammatory factors (Zhang et al., 2015). H_2_S promotes angiogenesis by inhibiting the formation of parstatin (protease-activated receptor-1, a fragment of PAR-1) and promoting VEGF activation, and significantly inhibits the degree of MI injury in male mice that were ligated to the left anterior descending (LAD) (Qipshidze et al., 2012). The novel H_2_S-donor 4-carboxyphenyl isothiocyanate (4-CPI) significantly attenuates myocardial infarct size and ventricular arrhythmias in isolated rat hearts (Testai et al., 2016). The mitochondria-specific H_2_S donor AP39 significantly reduced infarct size induced by myocardial I/R injury in rats by inhibiting mitochondrial permeability transition pore (PTP) opening and mitochondrial ROS production (Karwi et al., 2017).

Alpha lipoic acid prevents arrhythmia after cardiac I/R in rats by affecting K_ATP_ channels. This effect of alpha lipoic acid may be related to the release of sulfur sulfur and H_2_S (Dudek et al., 2014). The intake of beetroot juice (BRJ) prevents myocardial infarction and ventricular dysfunction after I/R in adult male CD-1 mice through CSE-mediated endogenous H_2_S production (Salloum et al., 2015). Na_2_S administration can reduce infarct size induced by myocardial I/R injury in db/db mice (Peake et al., 2013). Zofenopril reduces myocardial infarct size in mice and pigs after I/R injury by increasing H_2_S and NO (Donnarumma et al., 2016a).

In addition to protection against rat heart I/R damage, H_2_S has the effect of improving the adverse physiological changes caused by porcine aortic occlusion (Causey et al., 2015). Infusion of H_2_S could provide myocardial protection against Yorkshire boar myocardial infarct size induced by I/R injury by increasing the expression of phosphorylated p44/42 MAPK and decreasing Beclin-1 expression, as well as reducing cell necrosis (Osipov et al., 2009). NaHS also reduces myocardial infarct size in myocardial I/R rabbits via the cGMP/PKG pathway (Bibli et al., 2015). Except for studying animal models of cardiac I/R injury, in the hypoxia/reoxygenation injury model of cardiomyocytes, H_2_S could inhibit the hypoxia/reoxygenation-induced cardiomyocyte apoptosis of rat H9c2 cardiomyocytes by attenuating the endo/sarcoplasmic reticulum (ER/SR) stress (Li et al., 2015e). H_2_S also regulates autophagy through activation of mTOR (Xiao et al., 2015), regulates PI3K/SGK1/GSK3β signaling pathway (Jiang et al., 2016), down-regulates microRNA-1 (miR-1), and upregulates Bcl-2 to exert resistance in hypoxia-reoxygenation models of neonatal rat cardiomyocytes.

## Conclusion and Future Directions

**FIGURE 1:**
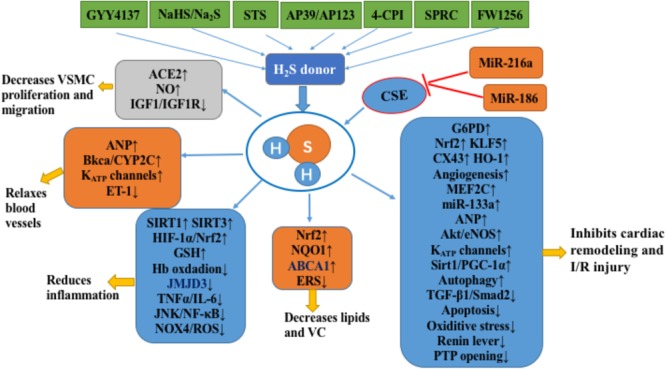
H_2_S in the cardiovascular system. H_2_S inhibits the occurrence and development of atherosclerosis by inhibiting the proliferation and migration of VSMCs, relaxing blood vessels, inhibiting inflammation, and inhibiting lipid accumulation and vascular calcification. In addition, H_2_S inhibits cardiac remodeling and cardiac ischemia-reperfusion injury by improving energy metabolism, anti-oxidation, anti-apoptosis, angiogenesis, and enhancing autophagy. The notation ↑ indicates increase or activation, and ↓indicates decrease or suppression. Abbreviations: STS, sodium thiosulfate; 4-CPI, 4-carboxyphenyl isothiocyanate; SPRC, S-propargyl-cysteine (also called ZYZ-802), a novel slow release H_2_S release compound (FW1256); VSMCs, vascular smooth muscle cells; ACE2, angiotensin converting enzyme 2; IGF-1, insulin-like growth factor-1; ANP, atrial natriuretic peptide; BKCa, large-conductance Ca^(2+)^-activated potassium channel; Cyp2C, cytochrome P-450 2C; K_ATP_, ATP-sensitive potassium channel; ET-1, endothelin-1; Hb, hemoglobin; JMJD3, Jumonji domain protein 3; TNFα, tumor necrosis factor alpha; IL6, interleukin 6′; JNK, c-Jun NH2-terminal kinase; NF-κB, nuclear factor-kappa B; Nox4, NADPH oxidase member NADPH oxidase 4; ROS, reactive oxygen species; Sirt1, sirtuin 1; Sirt3, sirtuin 3; HIF-1α, hypoxia-inducible factor 1-alpha; GSH, glutathione; NRF2, nuclear factor (erythrocyte-derived 2)-like 2; NQO1, NADPH dehydrogenase 1; ABCA1, ATP-binding cassette transporter A1; ERS, endoplasmic reticulum stress; VC, vascular calcification; G6PD, glucose-6-phosphate dehydrogenase; KLF5, Krüppel-like factor 5; Cx43, connexin 43; HO-1, heme oxygenase-1; MEF2C, myosin enhancer factor-2c; Akt, protein kinase B; eNOS, endothelial nitric oxide synthase; PGC-1α, peroxisome proliferator-activated receptor gamma coactivator 1 alpha; I/R, ischemia/reperfusion; CSE, cystathionine gamma-lyase; PTP, mitochondrial permeability transition pore.

In summary, H_2_S plays a significant protective role in atherosclerosis, cardiac hypertrophy, heart failure, and myocardial ischemia. Mechanisms responsible for these protective effects include down-regulation of oxidative stress responses, restoration of mitochondrial function, regulation of autophagy, attenuation of apoptosis, and increased blood vessel growth and angiogenesis (**Figure 1**). However, the evidence for these protective effects comes primarily from animal and cell models and lacks strong clinical evidence. The most urgent task for the future may be to replace H_2_S cardiac protection research from the laboratory to the clinic. In addition, plasma H_2_S levels in patients with cardiovascular diseases such as ACS and atherosclerosis are significantly reduced, which may provide strong support for clinical trials of H_2_S (Ali et al., 2016; Kanagy et al., 2017).

Donor research that restores the body’s reduced H_2_S to physiological level is an important area for future research. For example, SG-1002 is a long-acting H_2_S prodrug to restore as much as possible the reduction of H_2_S levels in the mouse heart failure model (Kondo et al., 2013). As a potential treatment for reversing H_2_S deficiency in heart failure, it has now entered clinical research (Polhemus et al., 2015). Various drug-H_2_S hybrids have been synthesized to enhance drug efficacy and/or reduce adverse drug reactions, which is also a future research direction. For example, sulindac is a chemopreventive agent and its gastrointestinal side effects are common. NOSH-sulindac releases NO and H_2_S and inhibits 12 human cancer cell lines from 6 different tissue sources, and is 1,000 to 9,000 times more potent than sulindac. In addition, compared with sulindac, NOSH-sulindac significantly increased gastrointestinal safety in rats (Kashfi et al., 2015).

It has been mentioned previously that the role of H_2_S in platelet aggregation is controversial, and the role of H_2_S in inflammation is also controversial. For example, C/EBP homologous protein (CHOP) regulates the endoplasmic reticulum stress response and is up-regulated after cecal ligation and puncture (CLP). The CHOP gene knockout improves the survival rate after CLP. H_2_S plays a protective role by activating Nrf2 to inhibit the expression of CHOP in macrophages (Ferlito et al., 2014). Whereas male Swiss mice undergo CLP, H_2_S increases the levels of pro-inflammatory mediators through mechanisms involved in NF-κB activation and aggravates systemic inflammation in sepsis (Zhang H. et al., 2007). In addition, H_2_S circulates freely throughout the body, shuttles between different cells, and acts on a variety of cellular targets (Cirino et al., 2017; Yuan et al., 2017). Therefore, the role of H_2_S is not limited to cardiovascular, so the use of H_2_S donors need to consider its impact on the overall physiological and pathological, in order to avoid adverse reactions caused by H_2_S in some special circumstances.

In the end, due to the potential clinical concern of superphysiological (sub-millimolar to millimolar) concentration of H_2_S delivered by sulfide salts (such as NaHS, and Na2S), we can anticipate that more and more H_2_S-enriched natural products or synthetic compounds will be developed for cardiovascular therapeutics. These compounds are pharmacotherapeutically relevant in cardiovascular diseases and are hopeful to stimulate endogenous H_2_S production or release physiological concentrations of H_2_S in a sustainable manner (Whiteman et al., 2015). In addition, mitochondria-targeted donors have an >1000-fold increase in potency in inhibiting high glucose-induced endothelial damage (Gerő et al., 2016), and the mitochondria-specific H_2_S donor AP39 significantly reduced infarct size induced by myocardial I/R injury in rats (Karwi et al., 2017). Therefore, mitochondria-targeted H_2_S donors may represent an important direction of H_2_S research.

## Author Contributions

LZ and YW contributed to the writing of the manuscript. YL and LL contributed to the revision. XF, SL, and SX conceptualized the manuscript.

## Conflict of Interest Statement

The authors declare that the research was conducted in the absence of any commercial or financial relationships that could be construed as a potential conflict of interest.
